# Editorial: The impact of alcohol and drugs on suspects', victims' and witnesses' cognition and memory

**DOI:** 10.3389/fpsyg.2023.1209406

**Published:** 2023-05-24

**Authors:** Angelica V. Hagsand, Heather Flowe, Melanie Takarangi, Julie Gawrylowicz

**Affiliations:** ^1^Department of Psychology, University of Gothenburg, Gothenburg, Sweden; ^2^School of Psychology, University of Birmingham, Birmingham, United Kingdom; ^3^College of Education, Psychology and Social Work, Flinders University, Adelaide, SA, Australia; ^4^School of Psychology, Division of Psychology and Forensic Sciences, Abertay University, Dundee, United Kingdom

**Keywords:** alcohol, drugs, suspects, victims, eyewitnesses, cognition, memory, psychology

In many societies around the world, alcohol and other drug use and abuse pose major health and safety problems. There is a clear link between alcohol consumption and lack of impulse control and an increased risk of violence, which leads to a high prevalence of intoxicated victims, witnesses, and suspects—especially in relation to violent crimes. Although recent years have seen an increase in applied research on the acute effects of alcohol and other drugs on memory and cognition, especially on the topic of eyewitness memory, there are still many gaps in our knowledge. Indeed, more research is needed to further disentangle the often-complex effects of alcohol and other drugs, especially in applied forensic contexts, such as witness and suspect investigative interviewing.

For theoretical reasons, it is critical to understand how legal drugs (alcohol and tobacco) and illegal drugs/narcotics (e.g., cannabis, cocaine, and amphetamine) affect cognition (e.g., decision-making among suspects on how much information to share and when to confess or deny) and memory (e.g., victims' and witnesses' episodic long-term memory of a crime). For applied reasons, this research is crucial to develop evidence-based guidelines for legal practitioners—such as police, attorneys, prosecutors, and judges—on how to best support people who were acutely intoxicated before, during, or after the crime and/or while giving evidence and how to enable them to provide the most detailed and accurate accounts possible about their crime-related experiences. Ultimately, this scientific knowledge may help to resolve crimes and improve legal integrity in societies.

The six articles published on this Research Topic are one steppingstone to producing more scientific knowledge about this important issue. The goal is that this collection of articles contributes to narrowing the gap of knowledge in the field. We are excited that the articles on this Research Topic cover a variety of different issues, ranging from intoxicated witnesses and victims to suspects using a variety of research methods, such as laboratory experiments, archival methods, and online studies. We are also pleased that this collection of articles indeed is an international collection, with scientists from a wide range of countries such as Australia, Belgium, Iceland, the Netherlands, New Zeeland, Sweden, the United Kingdom, and the United States. There is evidence of the need for this research online; this Research Topic has attracted considerable attention already.

Bartlett et al. used an online survey to examine whether people were more or less likely to take on misleading information from a sober or drunk witness. In this study, sober participants viewed a mock crime involving either a sober or intoxicated witness, then judged the credibility of the witness' written statement (which included some misinformation) and answered questions about the crime. Participants rated intoxicated witnesses as less credible compared with sober ones but still included misinformation from these intoxicated witnesses in their own accounts about the mock crime. The study concludes that it is important to minimize the risk of co-witness influence before a police interview.

Hildebrand Karlén et al. carried out an archival study. They retrospectively analyzed collected police interviews from an alcohol-intoxicated key witness in the famous murder of Swedish Prime Minister Olof Palme. No one is yet sentenced in this case, despite the murder taking place in the mid-80s. The researchers found that it might be possible to retrospectively estimate the witness' range of intoxication for the point in time when the crime took place and use this knowledge to conclude how alcohol affected the memory of the witness in this case. The authors concluded that the key witness was most likely reliable in this case.

Some people consume alcohol during and/or after the event has happened, especially in response to coping with traumatic incidents. Butterworth et al. studied the impact of post-encoding alcohol intoxication on recall and remember-know judgments in a controlled lab experiment. Heavy drinkers were given alcoholic or non-alcoholic drinks *after* they witnessed a traumatic car accident. Immediately, and after 1 week, participants recalled what they could remember about the accident. While the alcohol and non-alcohol groups remembered a similar number of details, the alcohol group benefitted from being interviewed again after 1 week. The study concludes that a repeated delayed interview might be beneficial for intoxicated witnesses.

Stevens et al. also used a controlled lab experiment to examine the effect of low-to-moderate intoxication levels on female participants' memory for a computer-based hypothetical immersive dating scenario, where the participant imagined engaging in consensual and non-consensual sexual activity. There was no negative effect of alcohol on participants' memory of the different dating scenarios, and participants showed a high degree of accuracy in their recollections. The authors conclude that these findings may have important practical implications in police investigations involving intoxicated victims of sex crimes, and in particular, those victims with low-to-moderate intoxication levels may be reliable sources of information and should not be underestimated.

An archival study by Hagsand et al. examined Swedish police interrogations with suspects of low-stake crimes such as drunk driving and narcotics crimes. The key findings were that police officers used more confrontational interrogation techniques with suspects who were acutely intoxicated during the interrogation compared with sober suspects. In addition, suspects with substance use disorder were more cooperative and prone to confess than suspects without chronic alcohol/drug problems. The authors conclude that suspects displaying signs of intoxication or substance use disorder may be more vulnerable during police interrogations.

Otgaar et al. re-analyzed data from an already published meta-analysis to examine the raw mean difference in how alcohol affected people's memory. The goal of this study was to determine the smallest effect size of interest in expert testimony on alcohol and memory since it is common for memory scientists to appear in court as experts to assess the validity of intoxicated witness, victim, and suspect testimony. The authors conclude that even just an increase or a decrease in one detail, especially one incorrect detail, could be important when assessing an intoxicated person's testimony.

This collection of articles shows the magnitude of different research aims and different scientific methods used to address the research question of how alcohol and drugs affect witnesses, victims, and suspects' cognition and memory and highlights theoretical as well as applied implications. Future research should aim to test intoxicated people in more realistic field settings, such as bars, pubs, and clubs to examine the impact of moderate-to-high levels of intoxication on cognition and memory. Furthermore, police interviews and interrogations can be considered stressful and anxiety-provoking, and more research is required to study and disentangle the interaction between alcohol and stress on memory and cognition in applied settings. In addition, we also encourage scientists to continue the important study of analyzing real-world police interrogations and interviews using archival study methods.

## Author contributions

AH wrote the first draft of this manuscript. HF, MT, and JG gave valuable feedback and helped to revise the manuscript. All authors intellectually contributed to this editorial article and approved the submitted version for publication.

